# Nidogen-1/NID1 Function and Regulation during Progression and Metastasis of Colorectal Cancer

**DOI:** 10.3390/cancers15225316

**Published:** 2023-11-07

**Authors:** Matjaz Rokavec, Stephanie Jaeckel, Heiko Hermeking

**Affiliations:** 1 Experimental and Molecular Pathology, Institute of Pathology, Medical Faculty, Ludwig-Maximilians-Universität München, D-80337 Munich, Germany; 2 German Cancer Consortium (DKTK), Partner Site Munich, D-80336 Munich, Germany; 3 German Cancer Research Center (DKFZ), D-69129 Heidelberg, Germany

**Keywords:** NID1/Nidogen1, ITGAV, SNAIL, integrin, colorectal cancer, EMT, metastasis, progression

## Abstract

**Simple Summary:**

Nidogen1 (NID1) is a component of the extracellular matrix and basement membranes. NID1 represents a linker between laminins, collagens, proteoglycans and cell surface receptors to control cell polarization, migration, and invasion. In this article, we show that elevated expression of *NID1* mRNA and of the genes *ITGA3*, *ITGB1*, and *ITGAV,* which encode NID1 receptors, is associated with poor prognosis and advanced tumor stage of colorectal cancer (CRC) patients. Furthermore, we demonstrate the direct induction of the *NID1* gene by the transcription factor SNAIL/SNAI-1. In addition, we determined that NID1 promotes metastasis in a xenograft mouse model of CRC. Finally, we show that the NID1 receptor ITGAV represents a potential therapeutic target for CRC.

**Abstract:**

We have previously shown that the extracellular matrix and basement membrane protein Nidogen1 (NID1) is secreted by more malignant, mesenchymal-like CRC cells and induces the epithelial–mesenchymal transition (EMT) and promotes the migration and invasion of less malignant, epithelial-like CRC cells. Here, we performed a comprehensive bioinformatics analysis of multiple datasets derived from CRC patients and showed that elevated expression of *NID1* and the genes *ITGA3*, *ITGB1*, and *ITGAV,* which encode NID1 receptors, is associated with poor prognosis and advanced tumor stage. Accordingly, the expression of *NID1*, *ITGA3*, *ITGB1*, and *ITGAV* was associated with an EMT signature, which included SNAIL/SNAI1, an EMT-inducing transcription factor. In CRC cells, ectopic SNAIL expression induced NID1 and SNAIL occupancy was detected at an E-box upstream of the *NID1* transcription start site. Therefore, *NID1* represents a direct target of SNAIL. Ectopic expression of NID1 or treatment with NID1-containing medium endowed non-metastatic CRC cells with the capacity to form lung metastases after xenotransplantation into mice. Suppression of the NID1 receptor ITGAV decreased cell viability, particularly in CMS/consensus molecular subtype 4 CRC cells. Taken together, our results show that *NID1* is a direct target of EMT-TF SNAIL and is associated with and promotes CRC progression and metastasis. Furthermore, the NID1 receptor ITGAV represents a candidate therapeutic target in CMS4 colorectal tumors.

## 1. Introduction

CRC is the second most deadly cancer worldwide responsible for more than 900,000 deaths per year [1]. Metastasis accounts for over 90% of cancer mortality [2]. Therefore, a better understanding of the molecular and cellular basis of CRC metastasis is of high clinical relevance. Metastasis is a multistep process, which involves cancer cell invasion, intravasation into the bloodstream, extravasation to target organs, and outgrowth of metastatic lesions [3]. During metastasis, tumor cells acquire mesenchymal-like properties, which increase their migratory and invasive capacities and allow them to leave the site of the primary tumor [4,5]. The process by which cells switch from an epithelial to a mesenchymal cell state is known as EMT [6]. It has been suggested that there is extensive cross-talk between invasive and non-invasive cancer cells, as well as stromal cells from the tumor microenvironment [7,8].

Recently, we showed that factors secreted by mesenchymal-like, invasive CRC cells induce the EMT, migration, and invasion of epithelial-like, non-invasive, tumor cells [9]. This paracrine induction of EMT was mediated by Nidogen1 (NID1). NID1 is a component of the extracellular matrix and basement membranes [10]. The main function of NID1 is to provide a link between laminins, collagens, and proteoglycans and cell surface receptors to control cell polarization, migration, and invasion [11]. Interestingly, NID1 has previously been reported to have pro-tumorigenic effects in other tumor entities. For example, NID1 induces EMT and promotes chemoresistance in ovarian cancer [12]. Furthermore, NID1 promotes the formation of lung metastasis by breast cancer cells [13]. It has been shown that the inhibition of NID1 reduces endometrial tumor growth and metastasis [14]. Moreover, NID1 levels are elevated in plasma samples from ovarian cancer patients [11,15]. Previous studies showed that NID1 binds to the integrins αvβ3 and α3β1 [16,17]. These receptors are composed of dimers encoded by the genes *ITGAV*, *ITGB3*, *ITGA3*, and *ITGB1*. Integrins are signaling molecules that exist in different conformational states, which determine their affinity for extracellular matrix proteins [18]. Upon integrin activation, the focal adhesion kinase (FAK) is recruited and its auto-phosphorylation is followed by the subsequent recruitment and activation of SRC [19]. In addition, integrins also activate other pathways, such as the RAS–MAPK and PI3K–AKT signaling pathways [20]. Integrins have been implicated in almost every stage of cancer development from primary tumor formation to migration, invasion, and metastasis formation [21]. For example, it has been shown that cancer-associated fibroblasts promote CRC cell invasion via integrin αvβ3 [22]. However, the roles of NID1 and its integrin receptors in CRC are not well understood. Here, we investigated the role of NID1 and its integrin receptors in CRC progression and examined the regulation of *NID1* by the EMT-TF SNAIL. We demonstrate that NID1 promotes metastasis in a xenograft mouse model and is a direct target of SNAIL. Furthermore, we show that the NID1 receptor ITGAV represents a candidate therapeutic target for CRC.

## 2. Materials and Methods

### 2.1. Cell Culture, Treatments, and Transfections

The CRC cell lines DLD1, HCT15, HT29, SW480, and SW620 (ATCC, LGC Standards, Wesel, Germany) were maintained in the McCoy’ 5A medium (Thermo Fisher Scientific, Schwerte, Germany; #16600082) supplemented with 10% fetal bovine serum (FBS) (Thermo Fisher Scientific, Schwerte Germany; #A5256701) including 100 units/mL of penicillin and 0.1 mg/mL of streptomycin. All cells were cultivated in a 5% CO_2_-humidified incubator at 37 °C. DLD1 cells with stable expression of NID1 were described previously [9]. DLD1 cells with stable DOX-inducible expression of SNAIL (DLD1/pRTR-SNAIL) were described previously [17]. Doxycycline (DOX; Sigma, St Louis, MO; #D3447) was dissolved in water (100 μg/mL stock solution) and applied at a final concentration of 100 ng/mL. To maintain DLD1/pRTR-SNAIL cell pools, which harbor pRTR vectors, a final concentration of 8 μg/mL puromycin was used and changed to a fresh medium every two days. siRNAs (Thermo Fisher Silencer Select: negative control (#4611), NID1 (#s9554), ITGAV (#7570), and SNAIL (#s13185) were transfected with Lipofectamine RNAiMAX Transfection Reagent (Thermo Fisher Scientific, Schwerte, Germany; # 13778150) according to the manufacturer’s instructions.

### 2.2. RNA Extraction and Quantitative Real-Time Polymerase Chain Reaction (qPCR) Assay

A High Pure RNA Isolation Kit (Roche, Mannheim, Germany; #11828665001) was used to isolate total RNA according to the manufacturer’s instructions. A Verso cDNA synthesis kit (Thermo Fisher Scientific, Schwerte, Germany; AB1453A) was used to prepare cDNA from 500ng RNA. qPCR was conducted with Fast SYBR Green Master Mix (Thermo Fisher Scientific, Schwerte, Germany; 4385612) on the LightCycler 480 (Roche, Mannheim, Germany) system. The expression was calculated by the ΔΔCt approach with GAPDH serving as the internal reference for normalization. Each qPCR assay was carried out in triplicate. The sequences of qPCR primers were as follows: GAPDH (TGTTGCCATCAATGACCCCTT, CTCCACGACGTACTCAGCG), NID1 (ATCAGCAATCCTTGGCTCAC, CCTTGGGATTCCTCTGTTCA), and ITGAV (TGCTACCTCTGTGCCGC, GAAGAAACATCCGGGAAGACG).

### 2.3. Chromatin Immunoprecipitation (ChIP) Assay

Cross-linking of cells was performed with 1% formaldehyde (Merck) and stopped after 5 min via the addition of glycine at a final concentration of 0.125 M. Cells were harvested with SDS buffer (50 mM Tris pH 8.1, 0.5% SDS, 100 mM NaCl, 5 mM EDTA), pelleted, and resuspended in IP buffer (2 parts of SDS buffer and 1 part Triton dilution buffer (100 mM Tris-HCl pH 8.6, 100 mM NaCl, 5 mM EDTA, pH 8.0, 0.2% NaN3, 5.0% Triton X-100). Chromatin was sheered by sonication (HTU SONI 130, G. Heinemann) to generate DNA fragments with an average size of 700 bp. The following antibodies were used: Polyclonal SNAIL antibody (AF3639, R&D Systems, Minneapolis, MN, USA) and IgG control (#R-5506, Sigma, St Louis, MO, USA) for 16 h as previously described [23]. Washing and reversal of cross-linking were performed as described [23]. ChIP-DNA was analyzed by qPCR and the enrichment was expressed as % input. Experiments were performed in triplicate. The sequences of qChIP primers were as follows: NID1 (CCGCCTGATGACATCCCATT, GGGCCCCAAGTCATCAAAGA), 16q22 (CTACTCACTTATCCATCCAGGCTAC, ATTTCACACACTCAGACATCACAG).

### 2.4. CellTiter GLO Cell Viability Assay

Cells were seeded at a density of 10,000 cells per well in 96-well plates and transfected with siRNAs. After 48 h, cells were passaged 1:10 to new 96-well plates and transfected with siRNAs again. After 5 days, 100 μL of CellTiter GLO 2.0 solution (Promega, Walldorf, Germany; #G9241) per well was added and incubated for 15 min. Luminescence intensities were measured using an Orion II luminometer (Berthold, Bad Wildbad, Germany).

### 2.5. Metastases Formation in a Tail Vein Injection Xenograft Mouse Model

Control DLD1 cells, DLD1 cells stably expressing ectopic NID1 (DLD1/NID1), and DLD1 cells treated with NID1-containing conditional medium from DLD1/NID1 cells were injected into the lateral tail vein of 6–8-week-old male and female NOD/SCID mice (Charles River, Germany) using 25-gauge needles (2 × 10^6^ cells/0.2 mL HBSS). After eight weeks, mice were euthanized and their lungs were resected and examined for metastases using H&E staining. Briefly, lungs were embedded in paraffin. Next, 2 µm sections were prepared and placed on glass slides. Sections were deparaffinized with Xylene and stained with Hematoxylin and Eosin. Thereafter, sections were dehydrated and covered with a coverslip with VectaMount permanent mounting medium (Vector Laboratories, Burlingame, CA, USA). Animal experiments were approved by the Government of Upper Bavaria, Germany (55.2-2532.vet_02-18-57).

### 2.6. Bioinformatics Analysis of Public Datasets

All public CRC patient datasets used in this study are described in Table S1. Expression and clinical data of the TCGA rectal adenocarcinoma (READ) and colon adenocarcinoma (COAD) cohorts [24] were obtained from the MD Anderson standardized data browser (http://bioinformatics.mdanderson.org/TCGA/databrowser/ (accessed on 13 December 2018). For the analyses, the RNA-Seq by Expectation-Maximization (RSEM)-normalized expression values from the Illumina RNASeqV2 (genes) datasets were used. Clinical and expression data of other CRC patient datasets were downloaded from NCBI GEO (www.ncbi.nlm.nih.gov/geo (accessed on 14 July 2020)). The calculation of the differential expression between tumors and adjacent normal colon tissue was performed by a paired *t*-test. The calculation of the differential expression between tumors of different stages was performed using 1-way ANOVA with a post-test for a linear trend from stage 1 to stage 4. The hazard ratio and significance for survival analysis were calculated using the log-rank test. The Survminer R-package (https://CRAN.R-project.org/package=survminer (accessed on 5 April 2019)) was used to determine optimal cutoff values for the binary classification of cases (high/low expression). The CMS classification of public datasets was obtained from Guiney et al. [20]. CRC cell line characteristics and dependency datasets were retrieved from the Cancer Dependency Map (DepMap; https://depmap.org/portal/ (accessed on 28 May 2021)).

### 2.7. Statistical Analysis

Student’s *t*-test (two-tailed; unpaired) was used to calculate statistical differences between the two groups. The one-way analysis of variance (ANOVA) with the Tukey multiple comparison post-test was used to compare more than 2 groups. Fisher’s exact test was used to compare metastasis incidence. The Pearson correlation coefficient was calculated to evaluate correlations. *p*-values less than 0.05 were considered significant. In the figures, *p*-values are presented as: * *p* < 0.05, ** *p* < 0.01, *** *p* < 0.001.

## 3. Results

### 3.1. High Expression of NID1 and Its Receptors Is Associated with Poor Prognosis, Advanced Stage, and Mesenchymal Tumor Features in CRC

**Figure 1:**
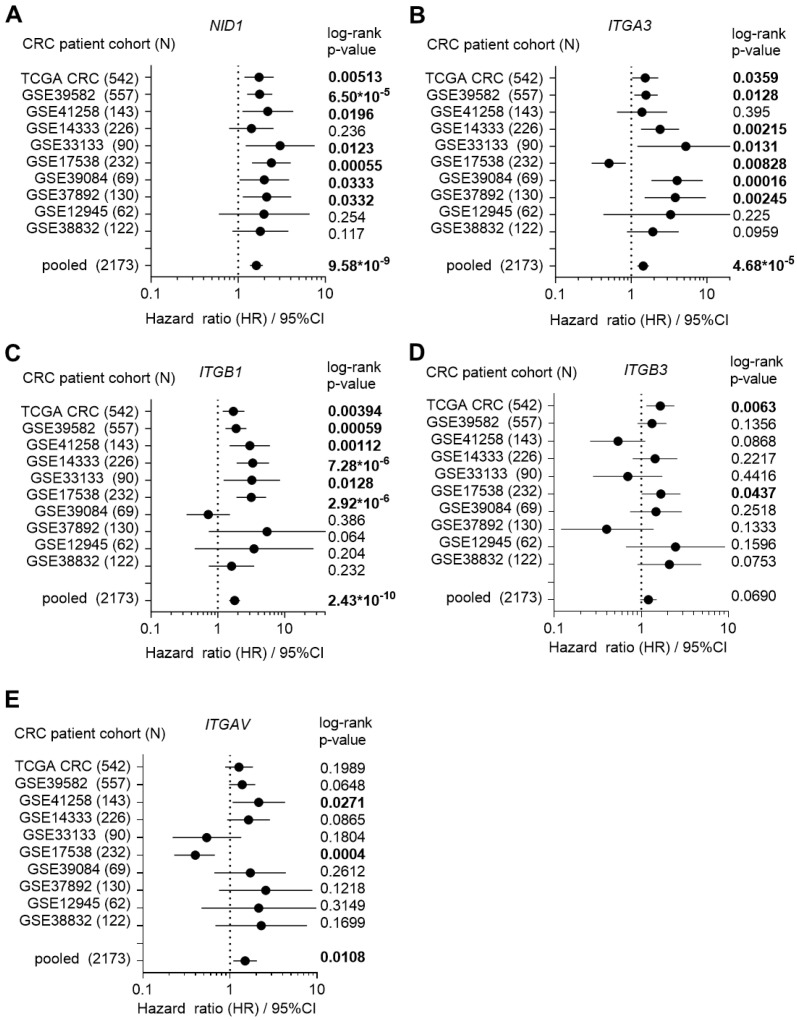
High expression of *NID1* and its receptors is associated with poor prognosis and advanced stage in CRC. (**A**–**E**) Forest plot showing Hazard ratios for relapse-free survival of indicated mRNAs by comparing patients with high versus low expression of indicated mRNAs in indicated CRC patient cohorts. Dots represent Hazard ratios and horizontal lines show 95% CI. *p*-values were calculated using the log-rank method. Significant *p*-values are highlighted in bold.

**Figure 2:**
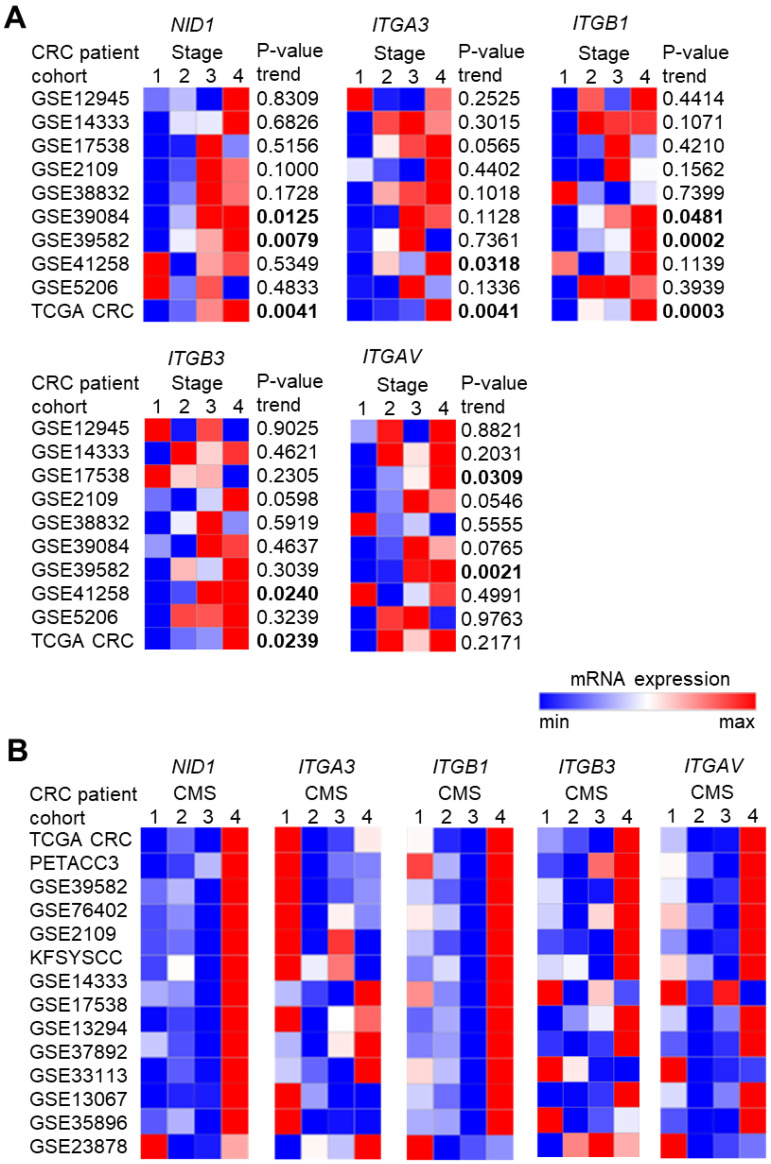
High expression of *NID1* and its receptors is associated with advanced-stage and mesenchymal tumor features in CRC. (**A**) Associations of the expression of indicated mRNAs with tumor stage. Significance was determined using one-way ANOVA with a post-test for linear trend from stages 1 to stage 4. (**B**) The expression of indicated mRNAs in different cancer molecular subtypes (CMS) in indicated CRC patient cohorts. Significant *p*-values are highlighted in bold.

**Figure 3:**
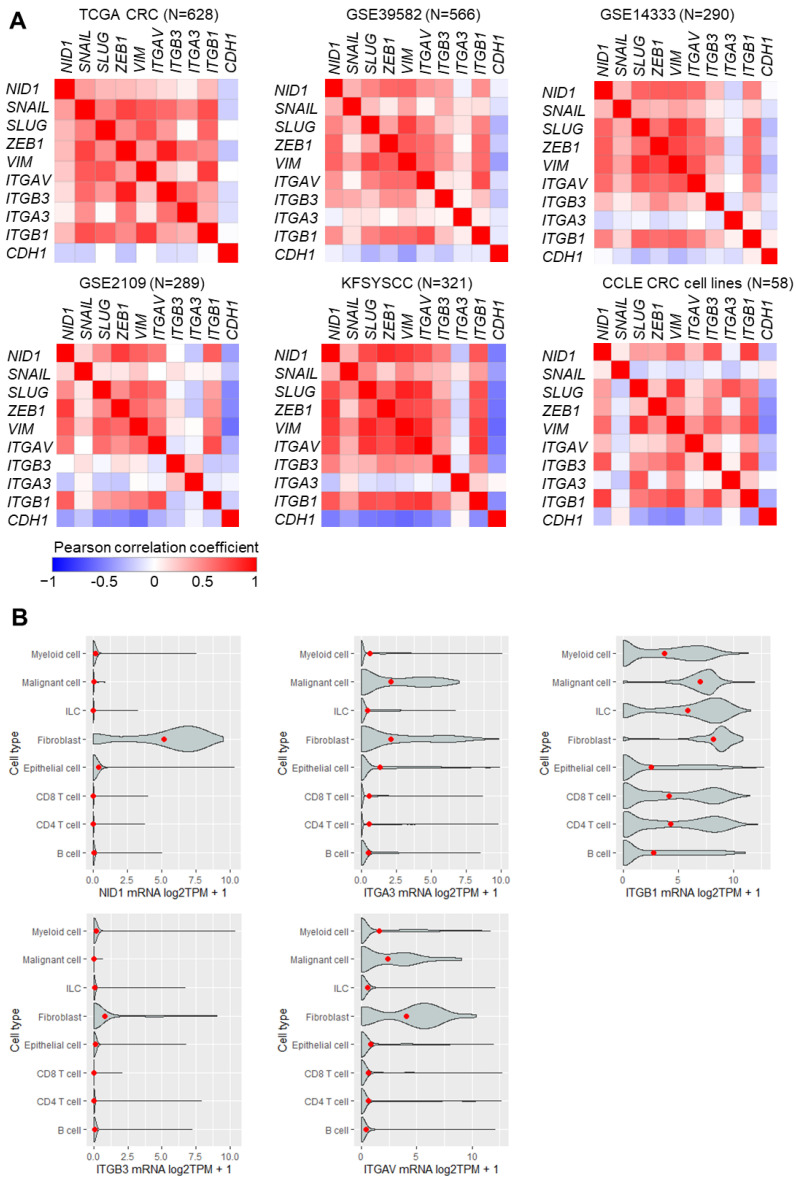
The expression of *NID1* and its receptors correlates with EMT-associated factors. (**A**) Correlation of the expression of *NID1*, its receptors, and EMT-associated mRNAs in primary tumors from indicated CRC patient cohorts. (**B**) Expression of indicated mRNAs in different cell types within colon tumors (data from single-cell RNA-seq (GSE81861)).

To investigate the role of NID1 and its receptors in CRC, we first analyzed the associations of *NID1*, *ITGA3*, *ITGB1*, ITGB3, and *ITGAV* mRNA expression with clinico-pathological parameters in publicly available CRC patient cohorts. High expression of *NID1* and its receptors was significantly and consistently associated with poor survival in the majority of 10 CRC patient cohorts (Figure 1A–E). Furthermore, the expression of *NID1* and its receptors progressively increased from stage 1 to stage 4 primary tumors in the majority of 10 CRC patient cohorts (Figure 2A). Next, we analyzed the expression of *NID1* and its receptors in different consensus molecular subtypes (CMS) of CRC [20]. The expression of *NID1*, *ITGB1*, *ITGB3*, and *ITGAV* was highest in the CMS4 subtype, which represents CRCs with mesenchymal features, metastasis, and poorest prognoses (Figure 2B). The expression of *NID1* correlated positively with the expression of *ITGAV*, *ITGB3*, and *ITGB1*, as well as with the mesenchymal-state-associated *SNAIL*, *SLUG*, *ZEB1*, and *VIM* mRNAs in expression profiles of five CRC patient cohorts, the CCLE CRC cell line panel, and in normal sigmoid and transverse colon tissue (Figure 3A and Figure S1). In contrast, *NID1* and the integrins correlated negatively with the expression of the epithelial-state-associated gene *CDH1* (Figure 3A and Figure S1). Finally, analysis of single-cell RNA-Seq data from primary colon tumors [23] showed that *NID1* and *ITGB3* mRNAs are predominantly expressed in fibroblasts, *ITGAV* and *ITGA3* are expressed in fibroblasts and cancer cells, and *ITGB1* is expressed in all cell types within colon tumors (Figure 3B). Altogether, these results show that elevated expression of *NID1* and its receptors is associated with poor prognosis, advanced stage, and mesenchymal tumor features, suggesting that NID1/integrin signaling may play an important role in CRC progression.

### 3.2. The Expression of NID1 Is Directly Induced by the EMT-TF SNAIL

**Figure 4:**
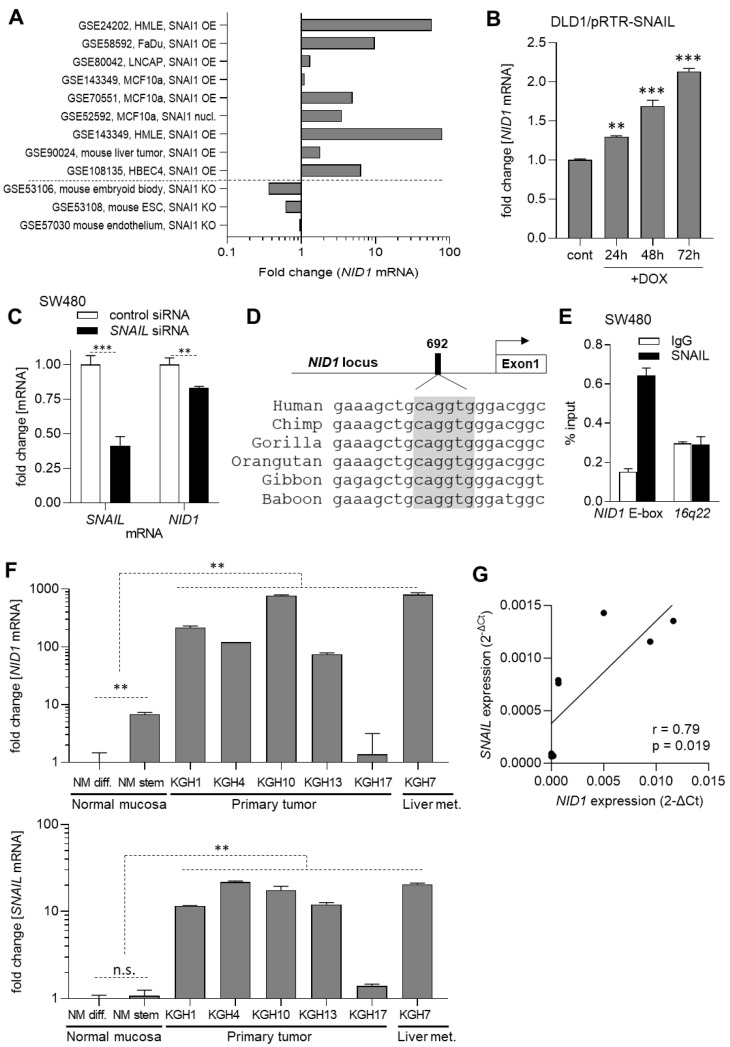
The expression of *NID1* is directly induced by SNAIL and elevated in CRC cells. (**A**) Fold changes in *NID1* expression in GEO datasets representing studies with SNAIL overexpression or knockdown in indicated cell lines. (**B**) qPCR analysis of *NID1* expression DLD1-pRTR/SNAIL cells after treatment with DOX for indicated periods. (**C**) qPCR analysis of *NID1* and *SNAIL* mRNA expression in SW480 cells after transfection with SNAIL siRNA for 48h. (**D**) Map of the human NID1 promoter region with the indicated conserved SNAIL binding site. SNAIL binding sequence motifs are indicated by grey shadowing. The arrow indicates the TSS. (**E**) qChIP analysis of SNAIL occupancy at the NID1 promoter and, as a control, the 16q22 locus in SW480 cells. (**F**) qPCR analysis of *NID1* and SNAIL expression in CRC patient-derived organoids. The groups were compared as indicated with the dotted lines. (**G**) Correlation between the expression of *NID1* and *SNAIL* mRNAs in CRC patient-derived organoids. NM diff.—organoids from normal colonic mucosa cultured in medium that induces differentiation; NM stem—organoids from normal colonic mucosa cultured in medium that maintains the self-renewal of colonic stem cells; KGH—tumor organoids. In panels (**B**,**C**,**E**,**F**), mean values ± SD are shown (*n* = 3). The groups were compared to the control group or to the group indicated by the dotted lines. ** *p* < 0.01, *** *p* < 0.001.

SNAIL, SLUG, ZEB1, and ZEB2 represent central EMT-inducing transcription factors. Since we observed a positive correlation between the expression of *NID1* and these factors, we asked whether the expression of *NID1* is regulated by them. Therefore, we first analyzed multiple public Gene Expression Omnibus (GEO) datasets obtained after ectopic expression or knockdown/knockout of *SNAIL*, *SLUG*, *ZEB1*, *or ZEB2* in cell lines or mice. *NID1* mRNA levels were induced after the ectopic expression of SNAIL and down-regulated after the repression of SNAIL in the majority of GEO studies (Figure 4A). On the other hand, NID1 expression was not consistently induced or repressed after the ectopic expression or knockdown/knockout of SLUG, ZEB1, or ZEB2 (Figure S2). Therefore, we focused on the regulation of *NID1* expression by SNAIL in subsequent analyses. Next, we utilized DLD1 CRC cells, which exhibit low levels of basal NID1 expression and harbor an episomal expression vector that confers conditional SNAIL expression [25]. Activation of ectopic SNAIL induced the expression of *NID1* in a time-dependent manner (Figure 4B). Conversely, the suppression of *SNAIL* by siRNA resulted in the repression of *NID1* expression in SW480 CRC cells, which show high levels of basal *SNAIL* and *NID1* expression (Figure 4C). When inspecting the *NID1* promoter, we identified a canonical SNAIL binding site 692 bp upstream of the TSS (Figure 4D). qChIP analysis showed an enrichment of SNAIL at the predicted SNAIL binding site in SW480 cells, thereby confirming the occupancy of SNAIL at the *NID1* promoter (Figure 4E).

### 3.3. Elevated Expression of NID1 in CRC Cells

Next, we analyzed the expression of *NID1* mRNA in a cohort of CRC patient-derived tumor organoids (PDTOs) and organoids derived from normal colonic mucosa, which was established previously [26,27]. In four out of five PDTOs, *NID1* expression was elevated when compared to organoids derived from normal colonic mucosa (Figure 4F). Furthermore, *NID1* expression was higher in organoids cultured in a medium, which maintains the self-renewal of colonic stem cells, when compared to organoids cultured in a medium that induces differentiation (Figure 4F). Finally, *NID1* mRNA levels were highest in the PDTO KGH7, which was derived from liver metastasis. The expression of *SNAIL* was significantly positively correlated with the expression of *NID1* within these samples (Figure 4G), consistent with *NID1* being a direct target gene of SNAIL. Altogether, *NID1* displays elevated expression in CRC cells when compared with normal colonic epithelial cells, which is masked by its elevated expression in other cell types of the tumor microenvironment.

### 3.4. NID1 Promotes Metastases Formation by CRC Cells

**Figure 5:**
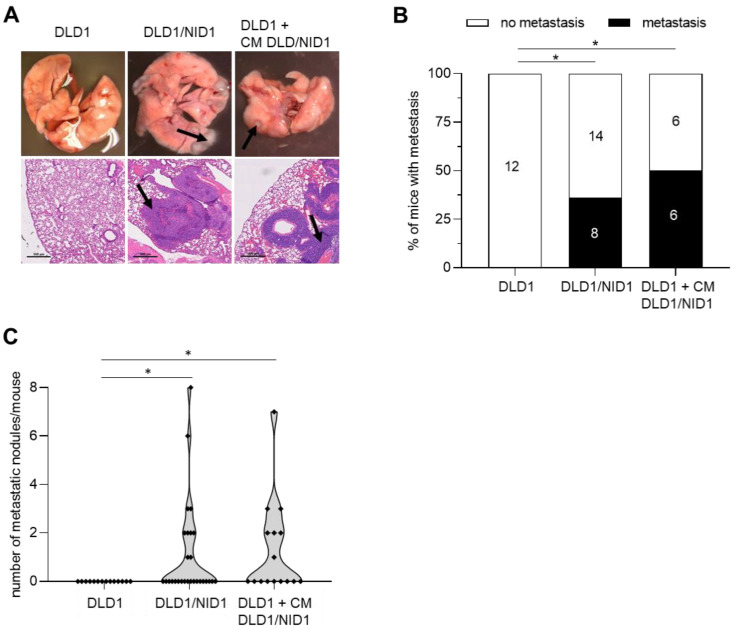
NID1 promotes metastasis formation of CRC cells. Formation of lung metastases by indicated cells, which were injected into tail-vein of NOD/SCID mice. (**A**) Upper: Representative lungs 8 weeks after tail vein injection. Lower: Representative images of H&E-stained resected lungs. Scale bar: 500 μm. Arrows indicate metastatic tumor nodules. (**B**) Incidence of metastasis in the lungs of indicated mice. (**C**) Quantification of metastatic nodules in the lungs of indicated mice. * *p* < 0.05.

We have previously shown that ectopic NID1 expression or treatment with NID1-containing conditioned medium induces EMT and enhances migration and invasion in DLD1 CRC cells [9]. Here, we performed mouse xenograft experiments to determine whether NID1 also increases the capacity of CRC cells to form lung metastases. Therefore, we injected control DLD1 cells, DLD1 cells stably expressing ectopic *NID1* (DLD1/NID1), which we previously generated [9], and DLD1 cells treated with an NID1-containing conditional medium from DLD1/NID1 cells into the tail veins of NOD/SCID mice. Eight weeks after injection, lungs were examined for metastasis formation. In total, 36% of mice injected with NID1-expressing cells showed lung metastases, whereas none of the mice transfected with a control vector formed lung metastases (Figure 5A–C). Interestingly, 50% of mice injected with DLD1 cells treated with the NID1-containing conditioned medium from DLD1/NID1 cells also showed lung metastasis (Figure 5A–C). In summary, our results show that endogenously produced and extracellular NID1 promotes the formation of metastases by otherwise non-metastatic CRC cells in mice.

### 3.5. Suppression of ITGAV Decreases Cell Viability of CRC Cells

**Figure 6:**
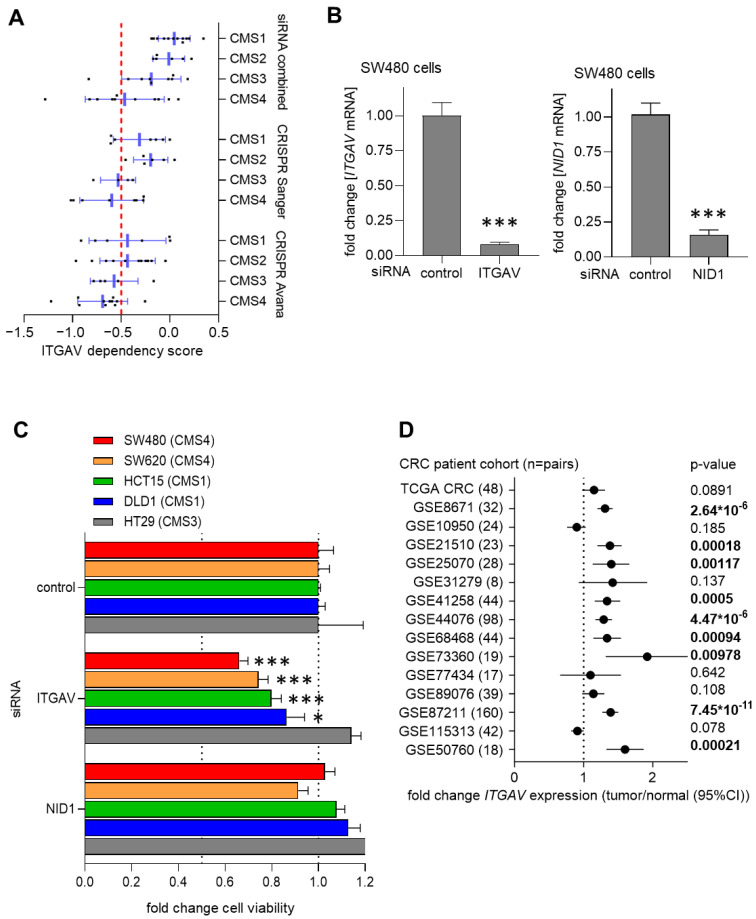
Suppression of ITGAV reduces cell viability. (**A**) ITGAV dependency score in CRC cell lines according to indicated CMS subtypes in three different screens. Data from the cancer dependency map. (**B**) qPCR analysis of *ITGAV* and *NID1* expression in SW480 cells after transfection with *ITGAV* or *NID1* siRNAs for 48 h. (**C**) Cell viability of indicated CRC cell lines transfected with indicated siRNAs for 1 week was determined by CellTiter GLO assays. (**D**) Forest plot showing fold changes in *ITGAV* expression between colorectal tumors and matched adjacent normal colonic mucosa in indicated patient cohorts. Dots represent fold changes and horizontal lines show 95% CI. Significance was determined using paired *t*-test. Significant *p*-values are highlighted in bold. In panels C and B, mean values ± SD are shown (*n* = 3). * *p* < 0.05, *** *p* < 0.001.

Finally, we asked whether NID1 or its receptors might represent therapeutic targets for the treatment of CRC. Therefore, we initially analyzed CRC cell line data from the Cancer Dependency Map (DepMap, [28]) database, which was obtained by genome-wide RNAi and CRISPR dependency screens in cell lines. CRC cell lines were not dependent on endogenous NID1, ITGA3, ITGB1, and ITGB3. However, CMS4 CRC cell lines particularly showed a dependency on ITGAV (Figure 6A). Cell lines with dependency scores of less than −0.5 for a particular gene are considered to be dependent on the expression of that gene [28]. To experimentally validate these findings, we suppressed the expression of *NID1* and *ITGAV* with siRNAs (Figure 6B). The suppression of *ITGAV* resulted in the repression of cell viability in four out of five CRC cell lines (Figure 6C). Notably, the decrease in cell viability was strongest in the CMS4 cell lines SW480 and SW620. The suppression of *NID1* did not significantly alter the cell viability of CRC cell lines (Figure 6C). Finally, the expression of *ITGAV* was consistently and significantly up-regulated in colorectal tumors when compared to matched adjacent normal tissue in 9 out of 15 publicly available CRC patient cohorts (Figure 6D), whereas no consistent up- or down-regulation of *NID1* expression was observed in colorectal tumors (Figure S3). Altogether, our results suggest that ITGAV represents a candidate therapeutic target, particularly for CMS4 CRCs, which are associated with metastasis and show the worst prognosis.

## 4. Discussion

In our previous study [9], we showed that NID1 promotes the migration and invasion of CRC cells. Here, we show that NID1 expression and/or addition to epithelial-like CRC cells is sufficient to endow them with the capacity to form lung metastases after injection into mice. NID1 is an integral component of basement membranes [10]. The interaction between tumor cells and basement membranes is crucial for cancer cell migration and invasion, which are necessary steps during metastasis [18]. Cell migration is mediated by the interactions between cell surface-bound integrins and the components of basement membranes. These interactions function as molecular clutches that propel the cell forward by converting actin polymerization at the protruding plasma membrane into traction force [29]. Our results suggest that more advanced tumors secrete high levels of NID1, which are incorporated into the basement membrane. Subsequently, cancer cells may interact with NID1 through cell-surface-bound integrin receptors, thereby promoting their migration. This process might also promote the migration of less advanced cancer cells that do not express NID1 but are able to interact with basement membrane-bound NID1, which was secreted from NID1-producing cancer cells. Altogether, these processes presumably promote collective cancer cell migration and invasion. Recently, it has been demonstrated that enteric neurons within the tumor microenvironment secrete NID1, which enhances CRC cell migration [30].

Here, we showed that the expression of *NID1* and its receptors is associated with poor survival and advanced tumor stage. According to data from the cancer dependency map, only the integrin ITGAV is relevant for the survival of CRC cell lines. Therefore, ITGAV may not only be involved in CRC progression but may also be important for the viability of CRC cells. Our results suggest that CMS4 subtype CRC cells are more dependent on ITGAV than CRC cells belonging to other subtypes. CMS4 CRCs showed the highest expression of ITGVA, which might explain the enhanced dependency of these cells on ITGAV. Consistently, a clinical study showed that combinational therapy with the anti-EGFR antibody Cetuximab and the anti-ITGAV antibody Abituzumab was particularly effective in patients whose tumors displayed high levels of integrin αvβ6 expression [31]. Integrin αvβ6 is involved in EMT and CRC metastasis [32,33]. Therefore, the inhibition of integrin ανβ6 might have the potential to target metastasizing tumor cells, which may explain the improvements in survival observed after Abituzumab treatment in patients with high-ανβ6-expressing tumors [31]. Patients with CRC belonging to the CMS4 subtype display the worst prognosis and the highest incidence of metastasis [34]. CMS4-CRCs are also the most difficult to treat since they lack the activation of targets of drugs that are currently used for targeted CRC therapy. On the other hand, the suppression of the ITGAV ligand NID1 did not affect cell viability. This is consistent with the DepMap data, which indicate that NID1 does not represent a vulnerability for CRC or other cancer types. In addition to NID1, ITGAV can also be activated by other ligands, including NRG1 [35], FGF1 [36], and IGF2 [37]. This may explain why the absence of NID1 does not completely abolish ITGAV signaling. Our results suggest that NID1 is involved in processes that promote CRC progression, such as EMT, migration, and invasion, whereas ITGAV is also essential for CRC cell viability.

Taken together, our results imply that NID1 contributes to CRC metastasis. Therefore, the suppression of NID1 might inhibit CRC progression and prevent metastasis. Furthermore, the NID1 receptor ITGAV might represent an attractive target for the treatment of CMS4 CRCs, as its inhibition decreased the viability of CRC cells. Here, we demonstrated that NID1 promotes metastasis in a mouse model, in which tumor cells are injected into the tail veins. Therefore, the early steps of metastasis, such as invasion and intravasation, were not recapitulated. In the future, genetic mouse models that recapitulate all steps of CRC metastasis should be utilized to study the role of NID1 in the early steps of metastasis.

## 5. Conclusions

In conclusion, we demonstrated that elevated expression of *NID1* and its receptors might represent potential markers for advanced CRC stage and poor survival. The direct regulation of *NID1* by SNAIL characterized here may explain the association of elevated *NID1* expression with advanced CRC. Since ectopic NID1 was sufficient to promote metastasis formation by CRC cells, the therapeutic inhibition of NID1 may abrogate CRC progression and prevent metastasis. In addition, the decrease in CMS4 CRC cell viability caused by inhibition of the NID1 receptor ITGAV implies that ITGAV represents a potential target for the treatment of CMS4 CRCs.

## Data Availability

The data presented in this study are available in this article and supplementary material.

## Supplementary Materials

The following supporting information can be downloaded at: https://www.mdpi.com/article/10.3390/cancers15225316/s1, Table S1. CRC patient cohorts used in the present study. The discrepancies in the number of patients are due to missing stage data in some of the cohorts; Figure S1. Correlation of the expression of *NID1*, its receptors, and EMT-associated mRNAs in normal sigmoid (A) and transverse (B) colon. Data are from GTEx; Figure S2. Fold changes in *NID1* expression in GEO datasets representing studies with SLUG (A) and ZEB1/2 (B) overexpression or knockdown/knockout in indicated cell lines or mice; Figure S3: Forest plot showing fold changes in *NID1* expression between colorectal tumors and matched adjacent normal colonic mucosa in indicated patient cohorts. Dots represent fold changes and horizontal lines show 95% CI. Significance was determined using paired *t*-test.
