# Supplementary material for: Nidogen-1/NID1 Function and Regulation during Progression and Metastasis of Colorectal Cancer
Source: Cancers (Basel). 2023 Nov 7;15(22):5316. doi: 10.3390/cancers15225316 (PMC10670298; doi:10.3390/cancers15225316)
Supplement: Supplementary file 1 [file cancers-15-05316-s001.zip › cancers-2665925-supplementary.pdf]

| CRC patient cohort | N | stage 1 | stage 2 | stage 3 | stage 4 |
|---|---|---|---|---|---|
| TCGA CRC | 627 | 90 | 196 | 144 | 80 |
| GSE39582 | 566 | 41 | 247 | 194 | 61 |
| GSE41258 | 182 | 28 | 48 | 49 | 57 |
| GSE14333 | 290 | 44 | 94 | 91 | 61 |
| GSE33113 | 90 | 0 | 90 | 0 | 0 |
| GSE17538 | 232 | 28 | 72 | 76 | 56 |
| GSE39084 | 69 | 8 | 23 | 16 | 22 |
| GSE37892 | 130 | NA | NA | NA | NA |
| GSE12945 | 62 | 13 | 23 | 21 | 5 |
| GSE38832 | 122 | 18 | 35 | 39 | 30 |
| GSE2109 | 290 | 39 | 88 | 78 | 45 |
| GSE5206 | 100 | 15 | 29 | 33 | 20 |
| PETACC3 | 688 | 0 | 108 | 580 | 0 |
| GSE76402 | 529 | NA | NA | NA | NA |
| KFSYSCC | 322 | NA | NA | NA | NA |
| GSE13294 | 155 | NA | NA | NA | NA |
| GSE13067 | 74 | NA | NA | NA | NA |
| GSE35896 | 62 | NA | NA | NA | NA |
| GSE23878 | 35 | NA | NA | NA | NA |

**Table S1.** CRC patient cohorts used in the present study. The discrepancies in the number of patients are due to missing stage data in some of the cohorts.

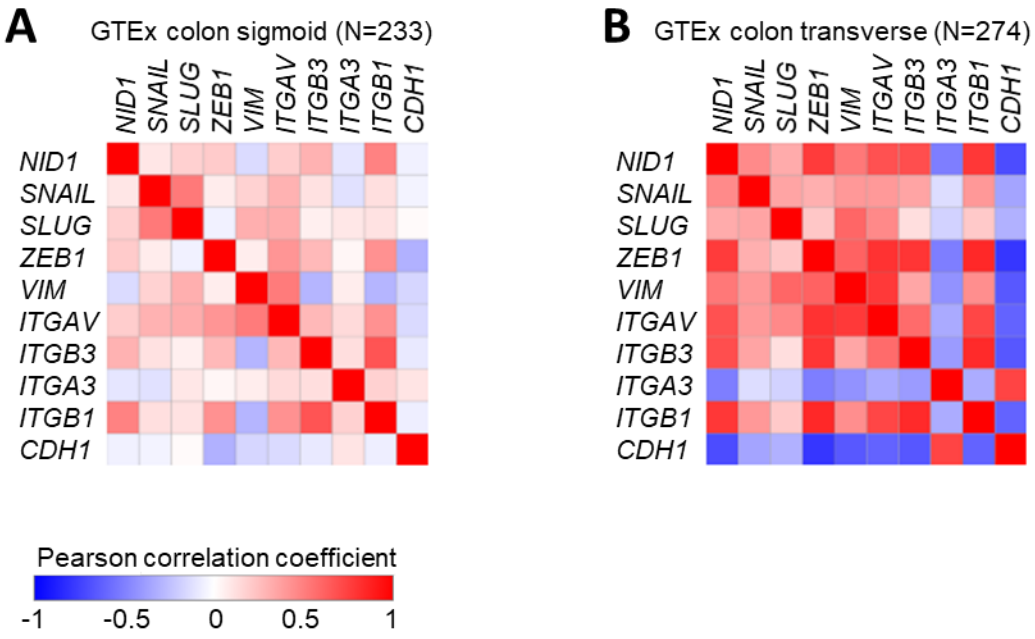


**Figure S1.** Correlation of the expression of *NID1*, its receptors, and EMT-associated mRNAs in normal sigmoid (A) and transverse (B) colon. Data is from GTEx.

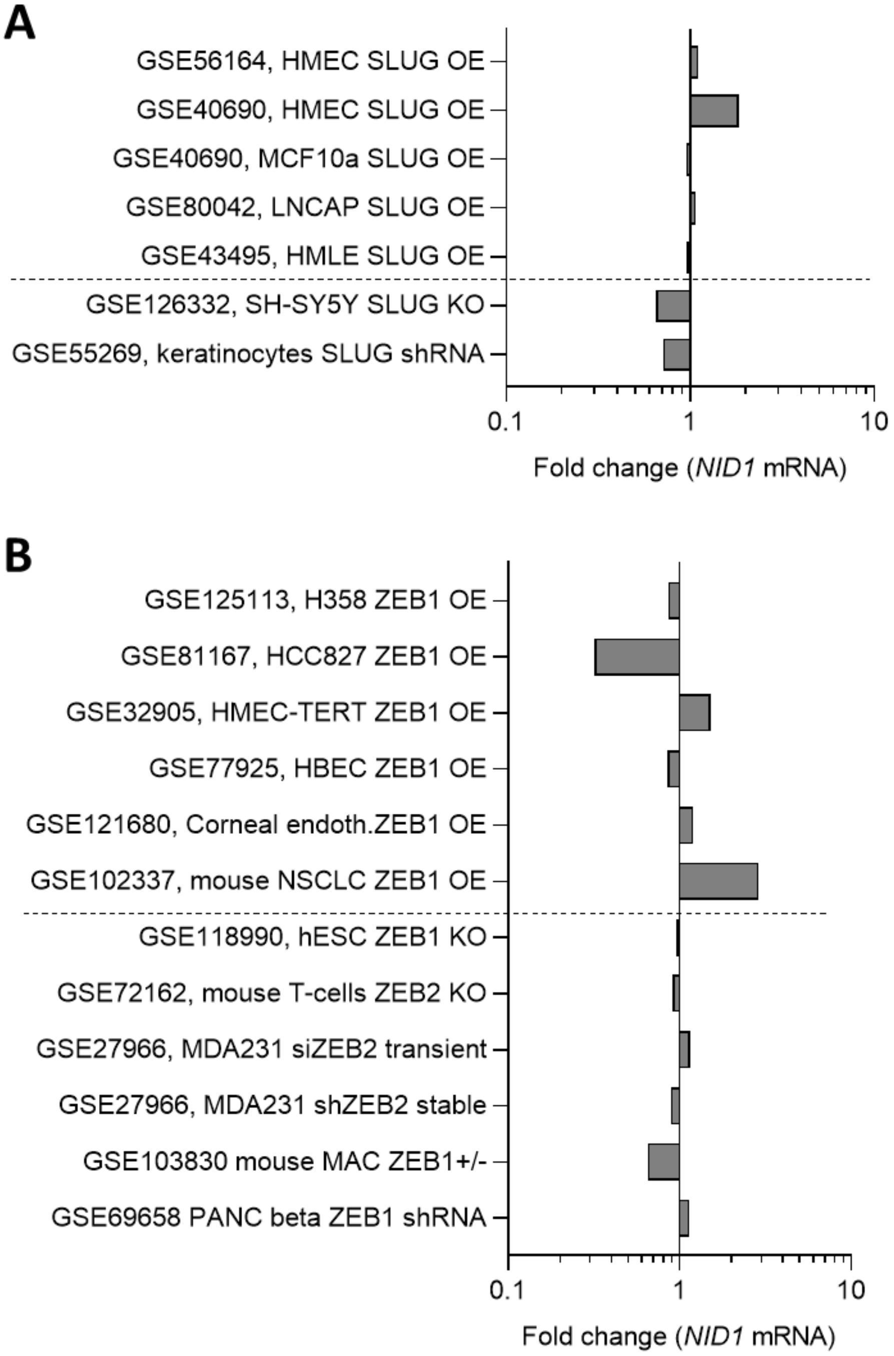


**Figure S2.** Fold changes in *NID1* expression in GEO datasets representing studies with SLUG **(A)** and ZEB1/2 **(B)** overexpression or knockdown/knockout in indicated cell lines or mice.

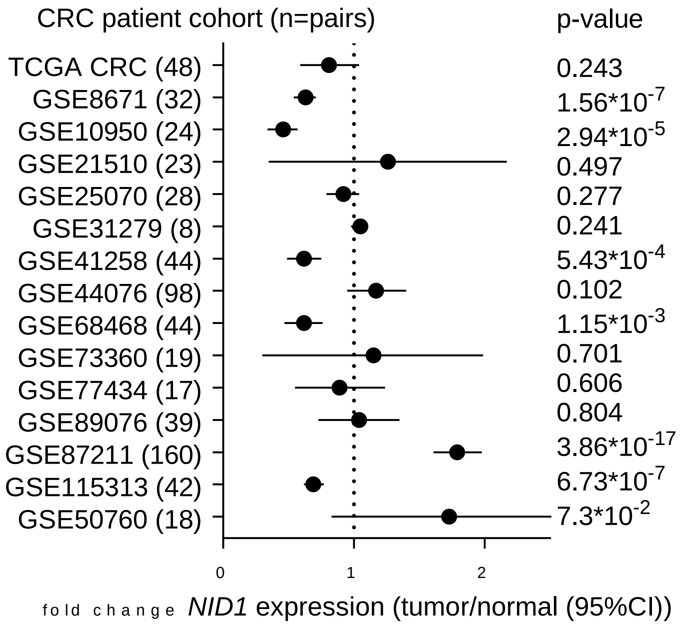


**Figure S3.** Forest plot showing fold changes in *NID1* expression between colorectal tumors and matched adjacent normal colonic mucosa in indicated patient cohorts. Dots represent fold changes and horizontal lines show 95% CI. Significance was determined using paired t-test.
